# The Study of Tropical Medicine

**Published:** 1906-09-01

**Authors:** 


### The Study of Tropical Medicine.

Diseases of warm climates require a very special course of study. The recent progress in these diseases has been so immense that the demand for instruction has necessitated the establishment of special schools, through which every candidate for colonial service shall pass before his appointment is finally approved.

*Diplomas and Degrees.*

It is now competent, for those who desire it, to obtain a University diploma in Tropical medicine, and this may be taken either at Cambridge or Liverpool. At Cambridge the fee is 6 guineas.

The Cambridge regulations give some idea of the ground to be covered for the diploma :—

> Any person whose name is on the Medical Register is admissible as a candidate to this examination, provided
>
> (I.) That a period of not less than twelve months have elapsed between his attainment of a registrable qualification and his admission to the examination;
>
> (II.) That he produce evidence, satisfactory to the Syndicate, that he has diligently studied pathology (including parasitology and bacteriology) in relation to tropical diseases, clinical medicine and surgery at a hospital for tropical diseases, and hygiene and methods of sanitation applicable to tropical climates.
>
> As evidence of study and attainments a candidate may present to the Syndicate (1) any dissertation, memoir, or other record of work carried out by himself on a subject connected with tropical medicine or hygiene; (2) any certificate or diploma in public health or sanitary science he may have obtained from a recognised examining body. Such evidence will be considered by the Syndicate in determining whether he is qualified for admission to the examination, and by the examiners in determining whether, if admitted, he shall be included in the list of successful candidates.
>
> The examination will have reference to the nature, incidence, prevention, and treatment of the epidemic and other diseases prevalent in tropical countries. It will comprise the following subjects :—
>
> 1. The methods of pathological and bacteriological investigation. The examination of the blood. The characters, diagnosis, and life-history of animal and vegetable parasites. The examination, chemical and microscopic, of poisonous or contaminated foods and waters.
>
> 2. The origin, pathology, propagation, distribution, prevention, symptoms, diagnosis, and treatment of the epidemic, endemic, and other diseases of tropical climates, including malaria; blackwater fever; trypanosomiasis; relapsing fever; dengue; yellow fever; plague; tetanus; beri-beri; dysentery and hepatic abscess, cholera, enteric fever, Malta fever, and specific diarrhœal affections of the tropics; diseases due to cestode and other worms; filariasis; bilharzial disease; specific boils, sores, and other cutaneous affections; mycetoma; ophthalmic affections of the tropics; affections caused by poisonous plants and animals, and by poisonous weapons; sunstroke.
>
> 3. The general effects on health in the tropics of seasons and climate, soil, water, and food; personal hygiene, acclimatisation; principles of general hygiene, with special reference to food supplies and water supplies, sites, dwellings, drainage, and the disposal of refuse; the sanitation of native quarters, camps, plantations, factories, hospitals, asylums, gaols, pilgrim and coolie ships; principles and methods of disinfection.

The University of Cambridge holds examinations in January and in August, to which any registered practitioner is admissible. Every candidate is required to pay a fee of £6 6s.

The University of Edinburgh grants a diploma in tropical medicine and hygiene. They must produce evidence of having attended approved courses of instruction in practical bacteriology, including the pathogenic micro-organisms of tropical diseases, in diseases of tropical climates, including the zoological characters and the life-history of disease-carrying insects, in tropical hygiene, and in the clinical study of cases of tropical disease, and certificates of proficiency in the methods of conducting *post-mortem* examinations and of preparing reports on them. The examinations are held in January and July. The fees for the first and any subsequent appearance are : Practical bacteriology, £1 1s.; diseases of tropical climates, £1 1s.; tropical hygiene, £1 1s.; tropical clinical medicine, £1 1s.; total, £4 4s.

The London School of Tropical Medicine.

The London School is situated near the Royal Victoria and Albert Docks, Woolwich, E., and is connected with the Branch Hospital of the Seamen's Hospital Society. The position of the school and hospital ensures a constant supply of clinical material, as the patients come from vessels trading with the tropics into the docks, which adjoin the hospital and school. Hence acute cases of many diseases peculiar to the tropics can always be studied, and adequate provision is made for clinical instruction by the hospital staff. The laboratory course occupies necessarily a prominent part of the curriculum, and includes daily practical teaching in the use of the microscope, the examination of the blood, and the recognition of the various parasites which are known to play so prominent a part in the production of tropical diseases. The full curriculum extends over three months, and the inclusive fee is 16 guineas. In each year there are three sessions, beginning on October 1, January 15, and May 1 respectively. Provision is made for the accommodation of a certain number of resident students, for whom also board is provided. This is a great advantage, as the curriculum is a very full one, and those who wish to pursue it thoroughly will be well advised to go into residence. Lastly, a word may be said of the help afforded by daily tutorial teaching given by a capable and experienced medical tutor. Applications for admission to the school should be made as early as possible, as the number of students desirous of coming into residence is greater than the school can accommodate.

Since the opening of the school 522 students have been in attendance, including 37 women graduates. The teaching is so arranged as to provide a complete course of instruction for the Cambridge diploma in tropical medicine.

### The Liverpool School of Tropical Medicine.

This school is carried on in conjunction with the University of Liverpool and the Royal Southern Hospital, for the purposes of research and clinical teaching respectively. A special ward at the hospital has been set apart for the reception and treatment of tropical diseases, and facilities for investigation and study are given in the Johnston Laboratories of the University.

Major Ronald Ross, C.B., F.R.S., F.R.C.S., is the Sir Alfred Jones Professor of Tropical Medicine and Consulting Physician at the hospital. At so important a port there is, of course, always a large seafaring population, and thus clinical opportunities are well represented. The fee for a full course (lasting about two months) is 10 guineas. A hall of residence is attached to the school.

In all instances both teaching and examinations are open to women as well as to men. At Liverpool there is a short course given to qualified nurses; the fee is 1 guinea.

### BOOKS FOR TROPICAL STUDY.

The following is a selection of the best books for the use of students of tropical medicine:—

1. "The Diseases of Warm Countries; A Handbook for Medical men." Scheube. Translated from the German by Pauline Falcke. Edited by Mr. Cantlie. 1903. 30s. net. J. Bale, Son and Company. Second Edition not translated.
2. "Tropical Diseases," by Sir Patrick Manson. Third Edition. 10s. 6d. net. Cassell and Company.
3. "The Medical Diseases of Egypt." Part 1. Dr. Sandwith. 7s. 6d. net. Henry Kimpton.
4. "Lectures on Tropical Diseases." Manson. 7s. 6d. net. Constable and Company.
5. "Les Filaires Du Sang De L'Homme." Pinnel. Not translated from the French. 6f. Rudeval.
6. "A Monograph of the Culicidæ of the World." Theobald. 3 volumes. (For advanced students.) £4 4s. British Museum, Longmans and Company, K. Paul and Company.
7. "Les Moustiques." Blanchard. Not translated from the French. 25f. Rudeval.
8. "Malarial Fevers." Thayer. 12s. net. H. Kimpton.
9. "Practical Study of Malaria." Stephens and Christophers. 12s. 6d. net. Liverpool School of Tropical Medicine. Williams and Norgate.
10. "Laboratory Studies in Tropical Medicine." Daniels. 15s. net. Bale, Son and Company.
11. "The Diseases of the Blood." Third Edition. Coles. 10s. 6d. net. J. and A. Churchill.
12. "Clinical Diagnostic Bacteriology." Coles. 8s. net. J. and A. Churchill.
13. "Animal Parasites in Man," by Dr. Max Braun (Bale, Sons, and Danielsson, Limited). Translated into English by Pauline Falcke. 21s.

